# Using Artificial Intelligence for Automatic Segmentation of CT Lung Images in Acute Respiratory Distress Syndrome

**DOI:** 10.3389/fphys.2021.676118

**Published:** 2021-09-14

**Authors:** Peter Herrmann, Mattia Busana, Massimo Cressoni, Joachim Lotz, Onnen Moerer, Leif Saager, Konrad Meissner, Michael Quintel, Luciano Gattinoni

**Affiliations:** ^1^Department of Anesthesiology, University Medical Center Göttingen, Göttingen, Germany; ^2^Unit of Radiology, IRCCS Policlinico San Donato, Milan, Italy; ^3^Institute for Diagnostic and Interventional Radiology, University Medical Center Göttingen, Göttingen, Germany; ^4^Department of Anesthesiology, DONAUISAR Klinikum Deggendorf, Deggendorf, Germany

**Keywords:** ARDS, fully automatic lung segmentation, deep learning, U-Net, LabVIEW, DeepLTK, Maluna, mechanical ventilation

## Abstract

Knowledge of gas volume, tissue mass and recruitability measured by the quantitative CT scan analysis (CT-qa) is important when setting the mechanical ventilation in acute respiratory distress syndrome (ARDS). Yet, the manual segmentation of the lung requires a considerable workload. Our goal was to provide an automatic, clinically applicable and reliable lung segmentation procedure. Therefore, a convolutional neural network (CNN) was used to train an artificial intelligence (AI) algorithm on 15 healthy subjects (1,302 slices), 100 ARDS patients (12,279 slices), and 20 COVID-19 (1,817 slices). Eighty percent of this populations was used for training, 20% for testing. The AI and manual segmentation at slice level were compared by intersection over union (IoU). The CT-qa variables were compared by regression and Bland Altman analysis. The AI-segmentation of a single patient required 5–10 s vs. 1–2 h of the manual. At slice level, the algorithm showed on the test set an IOU across all CT slices of 91.3 ± 10.0, 85.2 ± 13.9, and 84.7 ± 14.0%, and across all lung volumes of 96.3 ± 0.6, 88.9 ± 3.1, and 86.3 ± 6.5% for normal lungs, ARDS and COVID-19, respectively, with a U-shape in the performance: better in the lung middle region, worse at the apex and base. At patient level, on the test set, the total lung volume measured by AI and manual segmentation had a *R*^2^ of 0.99 and a bias −9.8 ml [CI: +56.0/−75.7 ml]. The recruitability measured with manual and AI-segmentation, as change in non-aerated tissue fraction had a bias of +0.3% [CI: +6.2/−5.5%] and −0.5% [CI: +2.3/−3.3%] expressed as change in well-aerated tissue fraction. The AI-powered lung segmentation provided fast and clinically reliable results. It is able to segment the lungs of seriously ill ARDS patients fully automatically.

## Introduction

The quantitative analysis of lung tomography [quantitative CT scan analysis (CT-qa)] images has been used extensively for more than 20 years and has significantly improved our knowledge of the pathophysiology of the acute respiratory distress syndrome (ARDS; ARDS Definition Task Force et al., 2012). Indeed, with CT-qa we have clarified how densities are distributed in ARDS, advancing the concept of the “baby lung” (Gattinoni et al., 1987; Bone, 1993; Gattinoni and Pesenti, 2005), showing how densities redistribute in prone position (Gattinoni et al., 1991; Pelosi et al., 1998; Cornejo et al., 2013), and explaining the mechanisms by which positive end-expiratory pressure (PEEP) acts (Pelosi et al., 1994). Determining the change in the non-aerated tissue fraction at two end-expiratory pressure levels, i.e., 5 and 45 cmH_2_O, is considered the gold standard for assessing recruitment in ARDS (Gattinoni et al., 2006). We were recently able to show that CT-qa can also provide valuable information for the respiratory management of COVID-19 (Chiumello et al., 2020). A precise segmentation [the inclusion of a structure into a region of interest (ROI) for the subsequent analysis] of the lung is mandatory for a reliable CT-qa. The actual segmentation procedure in several hospitals required constisten manual intervention. The time requirement and need of expert personal has serious hindered a broder adoption of CT-qa in clinical practice.

The application of machine learning techniques to image processing is currently of rapidly growing interest in the medical community (Seo et al., 2020a). Artificial neural networks (ANN) are a subfield of machine learning in which the underlying mathematical algorithm simulates the organization of the brain. By doing so, increasingly complicated tasks, such as voice and face recognition, recommender systems etc., which, until recently were considered impossible for a machine, have become part of our everyday life. The term deep learning (DL) goes back to around 2006 (Hinton et al., 2006). LeCun et al. (2015) explained DL in detail in 2015. Goodfellow et al. (2018) published an excellent textbook on DL in 2018. DL uses ANNs with many hidden layers. The larger the amount of data, the better DL works. Certain neural Network architectures such as the so-called convolutional neural networks (CNNs) are used specifically for image recognition. DL with CNNs are very common in medicine today (Chartrand et al., 2017; Litjens et al., 2017; Suzuki, 2017; Yasaka et al., 2018; Currie et al., 2019; Chassagnon et al., 2020; Al-Fatlawi et al., 2021; Guimarães et al., 2021; Jünger et al., 2021; Schwartz et al., 2021; Sułot et al., 2021; Wang C. et al., 2021; Yi et al., 2021).

There are two interesting CNN architectures for image segmentation. The “SegNet” developed by Badrinarayanan et al. (2017) and the U-Net developed by Ronneberger et al. (2015). SegNet was developed as an efficient architecture for semantic pixel-wise segmentation. It is primarily developed to recognize and classify in street scenes, streets, sidewalks, buildings, cars and pedestrians. SegNet was already used for Medical Image Segmentation (Sravani, 2019; Almotairi et al., 2020; Hu et al., 2020; Lei et al., 2021). The U-Net, was mainly developed for segmenting neuronal structures in electron microscopic stacks and light microscopic cell and tissue sections and works very effectively with comparatively little training data. In recent years, U-Net has been used successfully in medicine to segment certain structures and organs in chest x-rays and CT images (Zhou et al., 2018; Alom et al., 2019; Dong et al., 2019; Jeong et al., 2019; Hojin et al., 2020; Seo et al., 2020b; Umapathy et al., 2020; Causey et al., 2021; Ghosh et al., 2021; Wang Z. et al., 2021; Yan and Zhang, 2021). Some working groups have also segmented lungs in the CT images (Skourt et al., 2018; Park et al., 2020; Zhou et al., 2020; Chen et al., 2021; Jalali et al., 2021; Kumar Singh et al., 2021; Qiblawey et al., 2021). In a current publication, in the course of the COVID-19 pandemic, lung CT image segmentation with SegNet and U-Net was compared with each other, whereby the lungs segmented better with U-Net (Saood and Hatem, 2021). Gerard et al. (2020, 2021) developed a multi-resolution 3D-SegNet-CNN for the segmentation of inflamed, fibrotic and also ARDS lungs from the CT. This very interesting model consists of a high-res and a low-res Network and showed very good results. Seg3DNet is a fully convolutional CNN, but it uses less memory than SegNet and U-Net and can therefore process 3D images. There are now several modifications of the U-Net, such as U-Net ++ (Zhou et al., 2018), Res-U-Net (Umapathy et al., 2020), Recurrent Res-U-Net (Alom et al., 2019), 3D U-Net (Park et al., 2020), and more. Hofmanninger et al. (2020) trained a U-Net with 231 clinical cases (231 volumes with 108,248 slices). This data set contained different pathologies, reconstruction kernels, slice thicknesses, etc. This 2D U-Net231 showed surprisingly good results in the lung series tested. Hofmanninger et al. were able to show that automatic lung segmentation in routine clinical imaging is primarily a problem of data diversity. This was a very interesting aspect for us because we are working with clinical data. Due to the complexity of Networks such as 3DSegNet or the U-Net modifications, we decided to implement the 2D-U-Net.

The successful application of DL to the segmentation process of CT lung images in ARDS would greatly increase the use of CT-qa. It would become available to clinical practice for monitoring relevant variables, such as the size of the lung, the severity of lung injury, hyperinflation, recruitability, differentiate atelectatic and consolidated tissue, and assess parenchymal homogeneity. We developed a DL algorithm, based on the graphical programming language LabVIEW to automatically and efficiently analyze and segment acutely injured lungs over the full spectrum of ARDS severity.

## Materials and Equipment

### Dataset Descriptions

**FIGURE 1:**
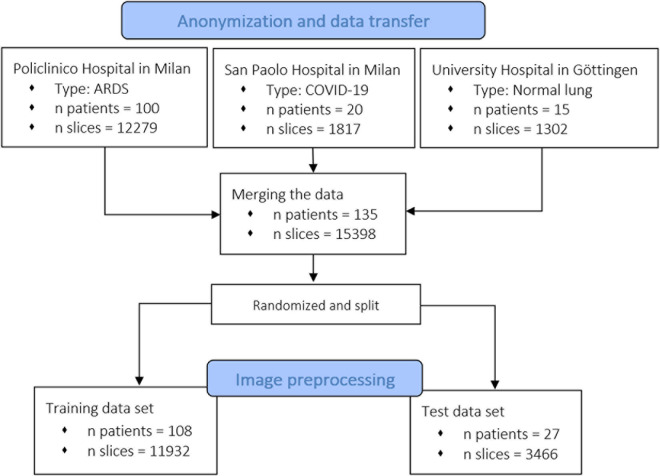
Flowchart of the inclusion of patients and related CT scans. We collected data from different centers, from healthy and acute respiratory distress syndrome (ARDS) lungs. As shown, the model was also trained and tested with recently acquired COVID-19 lungs.

The CT scan dataset used in this study (*n* patients = 100, *n* slices = 12,279) was extracted from an ARDS dataset in which we included the patients enrolled into different trials or physiological studies from 2003 to 2018 the Policlinico Hospital in Milan. The CTs of these ARDS patients were taken during an end inspiratory pause at 45 cm of water (recruitment) and at 5 and 15 cm of water during end expiratory pressure. The CT scan of these patients were performed within 4.1 ± 2.6 days after admission into the hospital. To this ARDS group, we added acquired CT scans from 20 COVID-19 patients (*n* slices = 1,817) from the San Paolo hospital, Milan and CT scans from 15 patients with normal lungs (*n* slices = 1,302) from the Medical University of Göttingen. From the included patients, we obtained 15,398 CT slices which were all manually segmented. We did not exclude any lung slice. Computed Tomography scans from eighty percent of the patients, randomly selected (*n* patients = 108, *n* slices = 11,932) were used for training of the algorithm and the remaining 20% (*n* patients = 27, *n* slices = 3,466) for the testing dataset. The characteristics of the dataset used in this study are summarized in Figure 1 and the characteristics of the patient population are presented in Table 1. The technical characteristics of the CT used to acquire the images are reported in Table 2. The ethics committee was notified and permission to use the data was granted (Göttingen Application Number 14/12/12).

**TABLE 1:** Summary of the datasets used to train and test the convolutional neural network.

	Train dataset		Test dataset		Sum	
			
	Patients (n°)	Slices (n°)	Patients (n°)	Slices (n°)	Patients (n°)	Slices (n°)
Normal lung	8	716	7	586	15	1,302
ARDS	89	10,222	11	2,057	100	12,279
COVID-19	11	994	9	823	20	1,817
Sum	108	11,932	27	3,466	135	15,398

**TABLE 2:** Technical characteristics of the CT scanner used.

Hospital	San Paolo Hospital Milan	Policlinico Hospital Milan	University Hospital Göttingen	
CT Scanner	GE Light Speed Qx/i	Siemens Somaton Definiton Flash	GE Lightspeed VCT	Siemens Sensation 16
KVP	120 kV, 140 kV	120 kV, 140 kV	140 kV	120 kV, 140 kV
Slice Thickness	2.0, 2.5, and 5.0 mm	5.0 mm	5.0 mm	5.0 mm
Pixel Spacing	0.6–0.8 mm	0.6–0.7 mm	0.6–0.7 mm	0.6–0.7 mm
Convolution Kernel	STANDARD	B30f, B31f, B40f	LUNG	B41f, B75f
Filter Type	BODY FILTER	0, FLAT, WEDGE_3	BODY FILTER	0
Patient Position	FFS, HFS	FFS	FFS	FFS, HFS

### Hardware and Software Used

We used a DELL Precision 5820 Tower with 32 GB RAM, a 3.70 GHz Intel (R) Xeon (R) W-2135 CPU and Windows 10 64-bit operating system. An Nvidia Quadro P 5000 with 16 GB GDDR5 RAM and 2560 CUDA cores was used as the graphics card. We used the same hardware for segmentation, quantitative analysis, and training. The U-Net was programmed with LabVIEW, NI-Vision (NI, Austin, TX, United States) and the Add-On Toolkit DeepLTK (Ngene, Yerevan, Armenia). There are many frameworks for DL (Caffe, Keras, TensorFlow, Theano, and Torch) on the market, but they mainly support the Python and C/C++ programming language. The DL toolkit, developed by Ngene, is a high abstraction level API providing the possibility to build, configure, train, evaluate and deploy deep neural Networks in the LabVIEW programming environment (Ngene, 2021). The GPU acceleration functionality of the toolkit is based on Nvidia’s CUDA and CUDNN toolkit, by calling corresponding shared libraries. CUDA is a parallel computing platform and programming model using a GPU for general purpose computing, and CUDNN is a GPU-accelerated library of primitives for deep neural Networks.

## Methods

### Image Preprocessing

**FIGURE 2:**
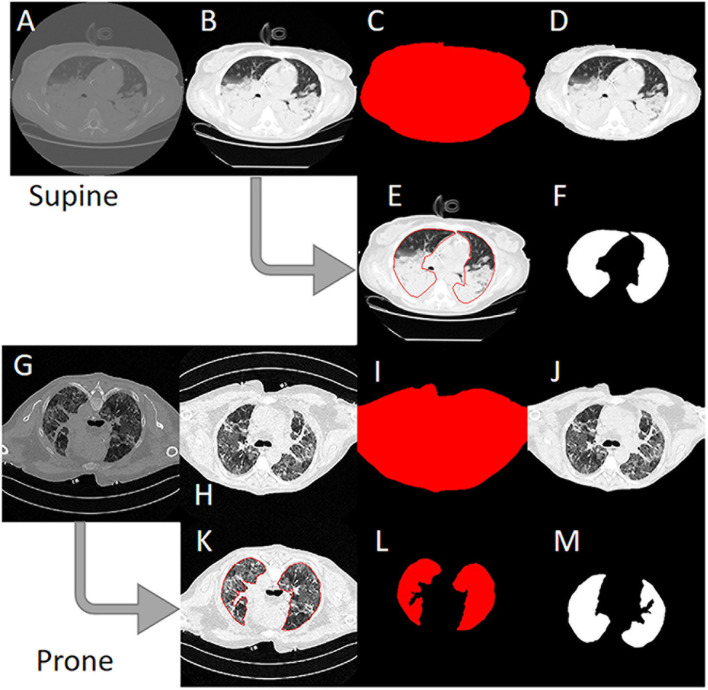
Image preprocessing steps to create the input data for the artificial neural network. **(A)** Original 16-bit gray value Digital Imaging and COmmunication in Medicine (DICOM) image. **(B)** 16-bit HU image. **(C)** 8-bit image. **(D)** Binary thorax mask. **(E)** Masked image. **(F)** Normalized image. **(G)** Manually drawn ROI. **(H)** Binary mask created from the region of interest (ROI). **(I)** Normalized lung mask (ground truth). **(K)** Rotated prone image. **(L)** Rotated prone mask.

Anonymized CT scans of the lungs obtained in the Policlinico Hospital Milan, San Paolo Hospital Milan and the University Hospital Göttingen were stored in DICOM (Digital Imaging and COmmunication in Medicine) format (^∗^.dcm) on DVD data carriers. In addition, a corresponding file with the coordinates of the manually drawn ROI was saved for each DICOM file (^∗^.xroi). These ^∗^.xroi files were created as follows. All reference segmentations (ground truth) were carried out manually and/or semi-automatically by experienced intensive care physicians using our own software (Maluna 3.14, Maluna 2020). The coordinates of these lung masks were then saved for each CT in a so-called ^∗^.xroi file (same file name as the Dicom image file name). They are loaded automatically when the DICOM image is loaded and placed as an ROI over the original image. Lung-specific calculations can be carried out within this ROI. Before the ANN could be trained with the lung CTs, the original DICOM images were preprocessed (Figure 2). In a first step, the gray values were converted into Hounsfield units (HUs) from the original 16-bit DICOM image (A,G) using the DICOM attributes “Rescale Intercept” (×0028, ×1,052) and “Rescale Slope” (×0028, ×1,053). The resulting image was then scaled to the range −1,024 HU to +100 HU and then converted into an 8-bit image (B,H). An 8-bit image is one with 256 levels of gray. A binary thorax mask was created (C, I) using a threshold and particle filter (Klapsing et al., 2017). Image B (H) was then masked in order to remove superfluous information from the image (D, J). In the final step, the pixels were normalized to the range between −1 and +1. This was done by subtracting 128 from each pixel and then dividing the result by 128.

To create the lung mask (known as “ground truth”), the ROI coordinates loaded from the ^∗^.xroi file drawn as an ROI in image E (K) are converted into a binary mask (F, L). Then, as with the lung image, the mask is normalized to the range −1 to +1 (I). For the CT images that were not obtained with the patient in the supine position, the images and masks were rotated accordingly (H, I M). To check the position of the patient, we used the DICOM attribute “patient position” (×0018, ×5,100). A total of 11,932 images and their manually generated ROI coordinates were preprocessed in this way and then loaded into the ANN. The image preprocessing was carried out with our own software Maluna 2020.

### The ANN

#### Structure of the ANN

**FIGURE 3:**
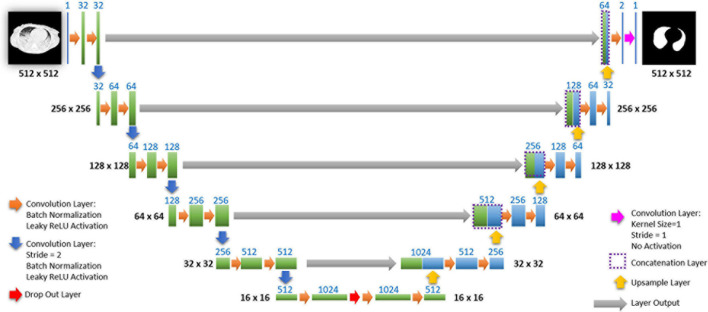
Our used U-Net architecture. Each green or blue box corresponds to a multi-channel feature map. The number of channels is shown above the box. The specifications 512 × 512 to 16 × 16 (in the lowest resolution) show the *x*, *y* dimensions in pixels of the input and output images (or feature maps).

The network we use is based on the U-Net architecture. The U-Net was programmed with the graphical programming language LabVIEW, with which we had many years of experience in the development of software for image analysis. The unique concept of U-Net is that it is able to generate a new, altered image as the output from an input image, after appropriate processing. This is very useful for generating segmentation images. The U-Net is a so-called fully convolutional network. Our U-Net programmed with LabVIEW is shown in Figure 3.

The architecture has a symmetric “U” shape and consists of two major parts: a contraction path (left side) and an expansion path (right side). The path follows the typical architecture of a convolution neural network. It consists of the repeated application of two convolution layers, each layer with batch normalization, followed by an activation function. In all convolution layers we use a filter kernel size of 3 × 3 pixels. For each convolution we used the so called “SAME” padding type, which means there is automatically enough padding that the output image of the convolution layer has the same dimensions as the input image.

In the original U-Net by Ronneberger, the image is filtered twice with 64 convolution filters in the first level of the contraction path. Due to insufficient graphics memory, we had to modify the original U-Net a bit. In the first level of the contraction path, the preprocessed lung CT image is therefore filtered twice with only 32 different convolution kernels. We used a filter kernel size of 3 × 3 pixels. A copy of this batch of 32 filtered images is transferred to the right part of the network. In the original U-Net, the next step is a max pooling layer for down sampling. We used a 3 × 3 convolution layer with stride 2, which halves the size of the input image (from 512 × 512 to 256 × 256 pixels) and doubled in the number of filtered images (from 32 to 64 images or channels). This principle, i.e., twice convolution filtering, halving the image size, and doubling the number of channels, is followed until we finally get a stack of 1,024 channels with a size of 16 × 16 pixels (this size is approximately in the range of an acinus). Since these small images no longer have any resemblance to the original image, but show certain extracted properties of the image, i.e., corners, edges, structures, they are also referred to as feature maps. These feature maps are processed further in the expansion path (right part) of the U-Net.

Each step on the expansion path consists of upsampling the feature map, followed by a convolution layer (“up-convolution”) which halves the number of feature channels and doubles the size of the input image. Then, a concatenation is carried out with the corresponding feature map from the contraction path (left part of the U-Net) followed by two convolution layers with batch normalization and an activation function. In the lowest path with the lowest resolution (16 × 16 pixels, 1,024 feature channels) a drop-out layer (with probability set to 0.4) was programmed between two convolutional layers.

Preprocessed lung CT-images are input to the contracting path, and lung mask predictions are output from a final layer following the expansive path. This final output layer is a 1 × 1 convolutional layer with no activation and a single filter. Batch normalization and dropout are proven methods of avoiding overfitting with CNNs (Srivastava et al., 2014; Ioffe and Szegedy, 2015).

#### Convolution Layers

The input image is first processed by a set number of convolution filters that have a fixed pixel size. In our case the size was 3 × 3 pixels. This filter then moves in a constant step size (stride) like a window from left to right over the pixels of the input image. After each pass, the filter skips to the next-lower row. The so-called padding is used to determine how the filter should behave when it hits the edge of the matrix. We use “SAME” padding. With “SAME” padding and Stride 1, the convolution layer output will have the same spatial dimensions as its input. With a 3 × 3 pixel filter, nine pixels of the input image are simultaneously connected to the filter (local connectivity) and are convolved to a new value.

The following equation shows the computation of the discrete convolution


(1) $$O\,\left[i,j\right]=\sum_{p=\frac{-S}{2}}^{\frac{S}{2}}\sum_{q=\frac{-S}{2}}^{\frac{S}{2}}\left[i-p,j-q\right]\cdot K\,\left[p,q\right]$$

(2) O=I⋅K⁢(c⁢o⁢n⁢v⁢o⁢l⁢u⁢t⁢i⁢o⁢n)

where *I* is the Input Image, *O* is the Output Image, *K* is the Filter Kernel, and *S* is the Filter Size.

**FIGURE 4:**
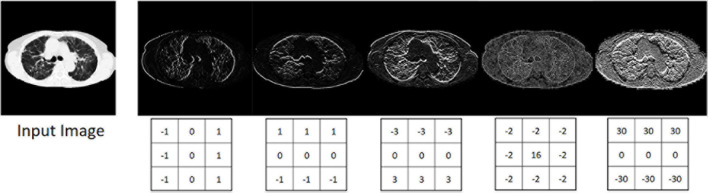
Input image and five different output images generated with different convolution kernels.

Depending on the property and number of filters, the convolution layer is able to recognize and extract individual features in the input data. These can be lines, edges or certain shapes (Figure 4). The step size of the filter determines whether the output image should have the same size as the input image, or whether it should be reduced in size. For example, for downsampling we chose a stride of 2 to halve the size of the input image. For upsampling we use an upsampling layer. This layer increases the dimensionality (rows and columns) of output feature maps by doubling the values (stride = 2).

#### Activation Function

In the activation function of the neural network, you decide whether the neuron fires or not. There are different types of activation functions such as sigmoid function, tangent function, rectified linear unit (ReLU) and leaky rectified linear unit (LReLU). In our case we used LReLU. LReLU s are one attempt to fix the “dying ReLU” problem. Instead of the function being zero when *x* < 0, a leaky ReLU will instead have a small negative slope like 0.1 or 0.3 (Maas et al., 2013; Goodfellow et al., 2018). That is, the function computes:


(3) f(x)=1(x<0)⋅(α⋅x)+1(x≥0)⋅(x)

where α is a small constant. So, if the input x is greater than 0, then the output is *x*. If the input is less than 0, the output will be alpha α times the input.

In the DeepLTK toolkit LReLU activation function uses 0.1 as a hardcoded alpha parameter.

#### Batch Normalization

Batch normalization is a layer that allows every layer of the network to perform learning more independently. Batch normalization can be used as a regularization strategy to avoid overfitting the model. The layer is added to the sequential model to standardize the input or the outputs. It can be used at several points between the layers of the model. It is often inserted just after defining the sequential model and after the convolution and pooling layers. Batch normalization is a technique that has been widely used over the years and has proven to be very effective in several DL tasks. It uses the mean and variance computed within a small data stack to normalize its features during activation (Ioffe and Szegedy, 2015).

#### Dropout Layer

Dropouts are the regularization technique that is used to prevent overfitting in the model. Dropouts are added to randomly switching some percentage of neurons of the network. When the neurons are switched off, the incoming and outgoing connections to those neurons are also switched off. This prevents units from co-adapting too much (Srivastava et al., 2014).

#### Initialization of the Weights in the ANN

With each pass through a layer, the variance should remain as constant as possible. This prevents the signal from increasing toward infinity or vanishing to zero. This means that the weights in the network must be initialized so that the variance for *x* and *y* remains the same. This initialization process is known as Xavier initialization (Glorot and Bengio, 2010). We use Xavier initialization for all the weights in our U-Net.

### Training of the U-Net

**FIGURE 5:**
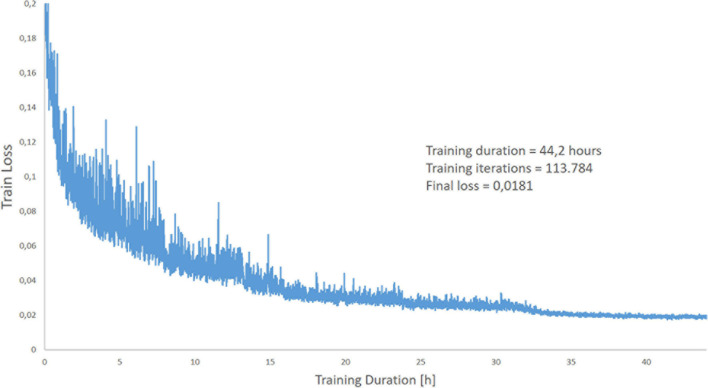
Graphical representation of the loss during the training period over 44.2 h.

**FIGURE 6:**
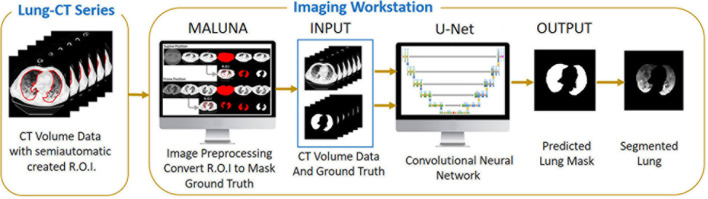
The complete training workflow.

The ANN programmed in this manner was trained with 11,932 CT slice images of lungs and the associated manually drawn lung masks (ground truth). The training was performed on 113,784 iterations. One iteration includes miniBatch sampling (we use a miniBatch size of 12) → Forward Propagation → Loss Evaluation (the predicted masks were compared to the manually generated lung masks) → Back Propagation and update of the weights in the network. In simple terms the network then tried to minimize the error between the manual mask and the mask generated in each iteration by selecting the appropriate combinations of convolution filters with more than 6,000 different convolution kernels used. This took about 1.4 s. The complete training duration was 44.2 h. Return values are the evaluated loss value and a value for the current iteration. We used the Mean Square Error Regression Loss function. The course of the learning curve is shown in Figure 5. The complete training process is shown in Figure 6.

### Testing of the Trained Model

The trained U-Net was tested on 3,466 CT lung slice images from 27 patients. The predicted ROIs were used to segment each slice and the whole lung CT-qa was then performed. Briefly, the lung is composed by two compartments with very different densities: tissue, with a density close to the one of water (0 HUs), and gas, with a density of −1,000 HU. For each voxel:


(4) $$V_{g\,a\,s}=\frac{-C\,T\,\left(H\,U\right)}{1,000}\cdot V_{v\,o\,x\,e\,l}$$

(5) $$\rho_{L}=\frac{C\,T+1,000}{1,000}$$

(6) $$T\,i\,s\,s\,u\,e\,m\,a\,s\,s=\rho_{L}\cdot V_{v\,o\,x\,e\,l}$$

The voxel gas volume and voxel tissue mass were multiplied by the total number of voxels to obtain the total tissue mass and the total gas volume. Lung tissue was classified according to its gas/tissue content as not inflated (CT number between +100 and −100), poorly aerated (CT number between −101 and −500), normally inflated (CT number between −501 and −900), and hyper-inflated (CT number between −901 and −1,000) (Cressoni et al., 2013).

We estimated recruitability as:


(7) $$R\,e\,c\,r\,u\,i\,t\,a\,b\,i\,l\,i\,t\,y=\frac{n\,o\,n\,a\,e\,r\,a\,t\,e\,d\,t\,i\,s\,s\,u\,e_{5\,c\,m\,H_{2}\,O}-n\,o\,n\,a\,e\,r\,a\,t\,e\,d\,t\,i\,s\,s\,u\,e_{45\,c\,m\,H_{2}\,O}}{n\,o\,n\,a\,e\,r\,a\,t\,e\,d\,t\,i\,s\,s\,u\,e_{5\,c\,m\,H_{2}\,O}}$$

**FIGURE 7:**
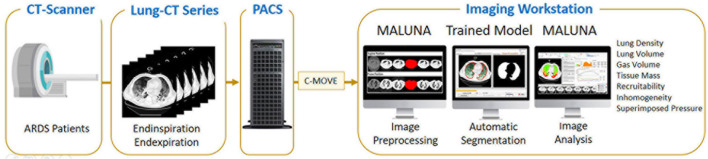
The complete test and analyzing workflow.

The first formula indicates the fraction of gasless tissue which regains inflation increasing the pressure. The complete testing workflow is shown in Figure 7.

### Statistical Analysis

The masks obtained by manual and artificial segmentation were compared by the intersection over nion (IoU) metric method. The variables computed by CT quantitative analysis after manual and artificial Intelligence (AI)-segmentation were compared by linear regression, and Bland-Altman analysis, and calculating 95% confidence intervals to evaluate the agreement between the masks. Student’s *t* test was used to test the difference between the means of normally distributed values. Otherwise, we used the Wilcoxon test. Two-tailed *p* values < 0.05 were considered statistically significant. All statistical analyses were performed using R 4.1 (The R Project for Statistical Computing).

#### Intersection Over Union Metric

The IoU, also known as the Jaccard index, is an established method for determining the segmentation quality of segmented images. It is used to quantify the correspondence between the manually created lung mask (ground truth) and the lung mask predicted by the trained model. The IoU metric measures the number of pixels common to the manually created masks and the prediction masks divided by the total number of pixels present in both masks. A value of 1 indicates a 100% agreement of the masks and 0 means no agreement (Nowozin, 2014).

The following equation shows the computation of the IoU:


(8) $$I\,o\,U=\frac{\left|A\cap B\right|}{\left|A\cup B\right|}$$

where A is the manual generated mask (ground truth) and B is the predicted mask.

## Results

### Slices-Level Performance

**FIGURE 8:**
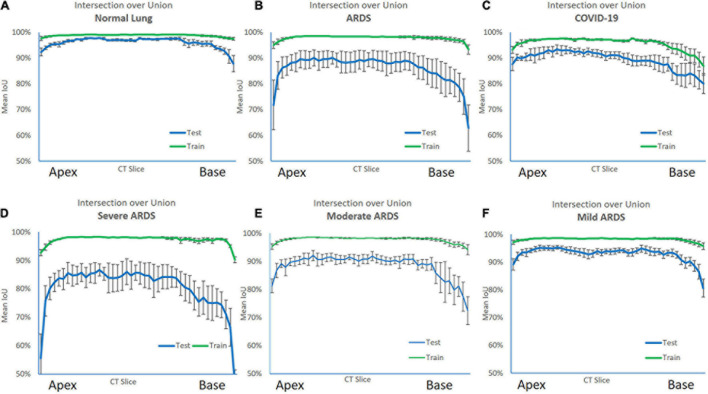
Intersection over Union (IoU) metric performance on the training (green line) and test set (blue line) along the cranio-caudal axis in normal lungs **(A)**, ARDS **(B)**, COVID-19 **(C)**, severe ARDS **(D)**, moderate ARDS **(E)**, and mild ARDS **(F)**. As shown, in the training set, the AI algorithm almost perfectly matched the manual segmentation. In the test set the performance was slightly poorer. The Figure also shows the anatomical distribution of the error. Indeed, the algorithm was able to achieve a higher performance in the middle of the lung, while at the apex and the base, locations where also for a trained eye is sometimes difficult to distinguish the lung parenchyma from the surrounding structures and the pleural effusion, it struggled more.

**FIGURE 9:**
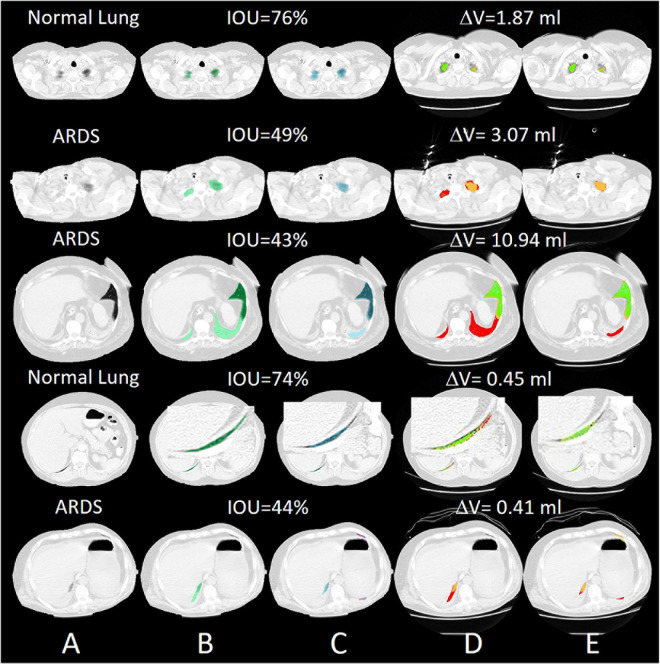
Selected slices with very poor segmentation results with an IOU of up to 43% in ARDS Lung. Most of the lungs were poorly recognized in the peripheral areas. **(A)** preprocessed CT, **(B)** ground truth (transparent green), **(C)** predicted mask (transparent blue), **(D)** quantitative calculations within the manual ROI and **(E)** quantitative calculations within the automatic ROI (green = normally aerated, blue = overextended, orange = badly aerated and red = not aerated).

**FIGURE 10:**
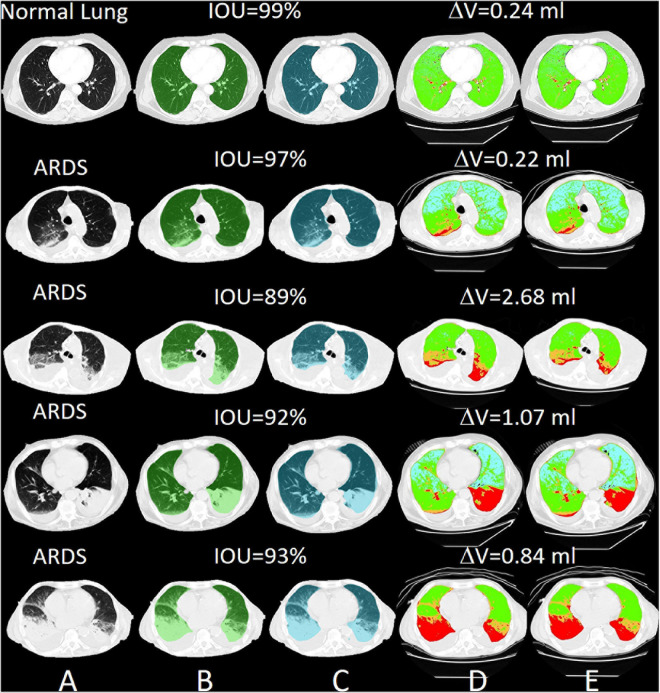
Selected slices with very good segmentation results with an IOU of up to 99% with normal lungs. The differences in lung volume between manually and automatically segmented lungs are 0.24 ml. **(A)** preprocessed CT, **(B)** ground truth (transparent green), **(C)** predicted mask (transparent blue), **(D)** quantitative calculations within the manual ROI and **(E)** quantitative calculations within the automatic ROI (green = normally aerated, blue = over-inflated, orange = poorly aerated and red = not aerated).

For all lung scans, the agreement between manual and AI-segmentation (IoU metric) was 87% ± 10% in the test set, as shown in Table 3. In Figure 8 we show the agreement between manually and AI-segmentation in the training and test sets along the cranio-caudal axis in normal lungs (Panel A), ARDS (Panel B), and COVID-19 (Panel C). Regardless of the lung type, the mean agreement between manual and AI segmentation across all CT slices was 91.3 ± 10.0, 85.2 ± 13.9, and 84.7 ± 14.0%, and across all lung volumes 96.3 ± 0.6, 88.9 ± 3.1, and 86.3 ± 6.5% for normal lungs, ARDS and COVID-19, respectively. In this test set, we found that the agreement between manual and AI-segmented lungs followed an inverse U-shape: higher in the central regions of the thorax and lower at the apex or near in the pleural recesses. Note that in these regions, the absolute amount of lung tissue is just a small fraction (4.1 ± 2.0%) of the entire parenchyma. The worst results were obtained in severe ARDS compared with moderate and mild ARDS (Figures 8D–F). Figure 9 shows the worst segmentation results, mainly in the peripheral zones of the lung slices. In addition to the IOU metric, the difference in lung volume between ground truth and predicted mask can also be seen. Figure 10 shows the best segmentation results with up to 99% agreement (IOU = 99%) in normal lungs.

**TABLE 3:** Mean Intersection over Union calculated per slices and per volumes.

	IoU_mean_ ± SD per slices	*N* Slices	IoU_mean_ ± SD per volumes	*N* volumes
Normal lung	91.3 ± 10.1%	586	96.3 ± 0.6%	9
ARDS	85,2 ± 13.9%	2,057	88.9 ± 3.1%	30
COVID-19	84,7 ± 14.0%	823	86.3 ± 6.5%	12
All lungs	87.3 ± 10.0%	3,466	89.6 ± 5.1%	51

### Patient-Level Performance

#### Lung Volume

The regressions and the Bland Altman analysis of the total lung volumes (gas + tissue volume) computed with manual and AI-segmentation both in the training set and in the test set are summarized in Supplementary Figure 6. As shown, the regression lines in these sets were close to identity. The Bland Altman plots on the sets showed biases of −3.1 ml [CI +13.0/−19.1] and −9.8 ml [CI: +56.0/−75.7 ml], respectively.

#### Lung Tissues

**FIGURE 11:**
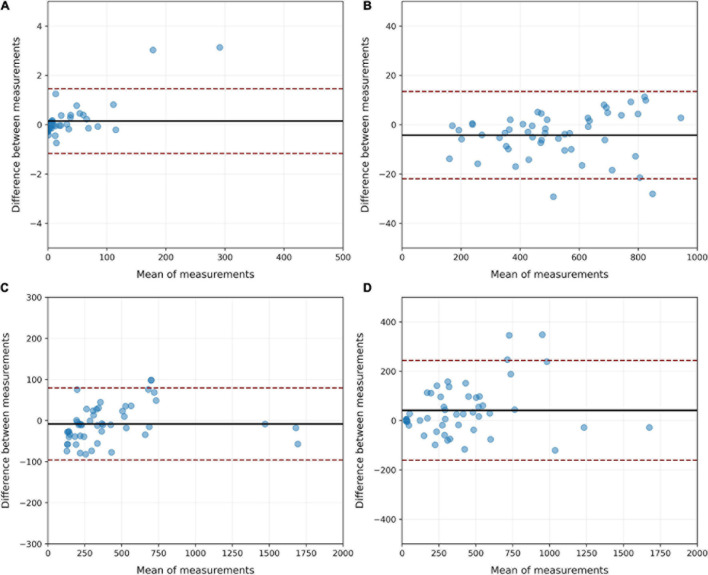
Bland Altman analysis of the agreement between manual and AI-segmentation on the test set when CT-qa was used to measure the over-inflated **(A)**, well-aerated **(B)**, poorly inflated **(C)** and non-aerated **(D)** tissues on the test set. As shown, biases never exceeded 50 g. The largest CI were, as expected, in the non-aerated tissue, where also for a trained eye is, at times, difficult to distinguish parenchyma from pleural effusion.

In the CT-qa, the overinflated, well-aerated, poorly aerated and non-aerated tissue fractions were almost identical in the manually or AI-segmentated images. Indeed, the *R*^2^ of the linear regressions between manual and AI-segmentation on overinflated, well-aerated and poorly aerated and non-aerated tissue was 0.99, 0.99, 0.98, and 0.91, respectively. The Bland Altman analyses comparing overinflated (*p* = 0.99), well-aerated (*p* = 0.91), poorly aerated (*p* = 0.91), and non-aerated tissues (*p* = 0.53) obtained after manual and AI-segmentation on the test set is summarized in Figure 11. The whole lung tissue fraction in all cases is 41.8 ± 17.2% in manual segmentation and 41.1 ± 16.2% (*p* = 0.85).

#### Recruitability

Assessment of recruitability is likely the most relevant variable that can be measured with CT-qa. In Supplementary Figure 7, we report the recruitment fraction computed for the manual and AI-segmented lungs in the test set. The recruitment is expressed both as variations of non-aerated tissue (panel A) and as a variation of well-aerated tissue (panel B). The agreement between the two techniques is within +6.2 and −5.5% (bias +0.3%) when the recruitment is expressed as variation of the percentage of non-aerated tissue and between +2.3 and −3.3% (bias −0.5) when expressed as variation of the percentage of well-aerated tissue.

#### Inaccuracies

To determine the inaccuracies of manual and AI-segmentation we assumed that the lung weight should not change in the same individual when increasing the airway pressure from 5 to 15 and to 45 cmH_2_O. A difference in lung weight between the two airway pressure levels can be considered as a sign of segmentation inaccuracy. As shown in Table 4, the average lung weight differences between 5 and 15 cmH_2_O or between 5 and 45 cmH_2_O obtained by manual and AI-segmentation were negligible. However, in the individual patients the differences could be as high as 336 g.

**TABLE 4:** Differences in lung weight detected at different airway pressures.

Train dataset				Test dataset			
	
		Manual	Automatic			Manual	Automatic
5–15 cmH_2_O	Mean (g)	–6.5	1.2	5–15 cmH_2_O	Mean (g)	17.2	–14.8
	SD (g)	64.7	60.5		SD (g)	83.9	54.3
	Min (g)	–227.5	–168.5		Min (g)	–103.0	–150.4
	Max (g)	173.2	196.2		Max (g)	194.8	42.6
5–45 cmH_2_O	Mean	7.5	14.5	5–45 cmH_2_O	Mean	39.8	–2.6
	SD (g)	93.8	92.6		SD (g)	92.1	72.4
	Min (g)	–226.0	–231.9		Min (g)	–94.8	–145.7
	Max (g)	336.0	339.8		Max (g)	150.7	101.8

#### Workload

The complete training of the neural Network up to the level used in this analysis lasted 44.2 h. The learning curve of the algorithm is reported in Figure 5. With our current configuration the analysis of an unknown single CT slice requires 0.041 ± 0.007 s. Therefore, automatic segmentation of a complete lung CT-scan with approximately 100 slices of 5.0 mm thickness, required approximately 5 s.

## Discussion

In this study, we found that automatic lung segmentation performed by a properly trained neural network provided lung contours in close agreement with the ones obtained by manual segmentation. When comparing lung CT slices with the original Ronneberger network (Hofmanninger et al., 2020, Table 3: test data set for lung slice only), the IOU of damaged lungs is in a similar range (85% vs. 80–87% for trauma and 85% vs. 83–91% for atelectasis). In the case of normal lungs, however, the results are worse in comparison (91% vs. 94%). The automatic approach completed segmentation of the entire lung in 5–10 s making immediately available the CT-qa. Therefore, the whole process from DICOM image extraction to the lung CT-qa with data on the fractions of inflated, well aerated, poorly aerated and non-aerated tissue, as well as lung recruitability can be completed in just a few minutes. Beyond their use for research, these data may prove important for the clinical diagnosis and respiratory therapy. Indeed, the greatest limitation in implementing CT-qa in the everyday clinical practice is the amount of man-hours required for lung segmentation. This study presents a possible solution to this problem. The trained model is not perfect, as it showed weaknesses in the edge regions of the apex and base, especially in severely damaged lung areas that are difficult to identify even for a trained radiologist. These represent, however, only a minor fraction of the total lung parenchyma that this should not exceed 10% of the entire lung mass. Overall, the results obtained are fully adequate for pathophysiological decision processes and consequent clinical application.

We may wonder to which extent one may be confident in the AI-segmentation compared to the manual one. In ARDS, image segmentation is especially difficult as, in some cases, it is almost impossible to discriminate the edge of the lung parenchyma from a pleural effusion, so common in ARDS (Chiumello et al., 2013), particularly in the most dependent lung regions and most severe ARDS forms. However, this problem is also present in manual segmentation. Indeed, when the CT scan of the same lung is taken under different operating conditions, for example, at different airway pressures, we observed, as in previous studies, differences in lung weights which, on average were rather trivial (∼10–20 g), but they could be as high as 336 g in the individual patient. These variations may result either from the segmentation procedure and/or from actual changes in lung weight, primarily due to a possible airway pressure-dependent blood shift. It is unfortunately impossible to determine how much of the weight variation is due to an intrathoracic blood shift or to inaccuracies of the segmentation process. The decrease in intrathoracic blood volume we estimated in a previous work (Chiumello et al., 2007) with increasing airway pressures was about 100 ml, leading to a small decrease in lung weight. In the present study, we found more pronounced variations of lung weight between 5 and 45 cmH_2_O than between 5 and 15 cmH_2_O. Indeed, especially in the train set, we found maximum differences in lung weight between the two pressure levels as large as 336 g, making unlikely that blood shift alone accounted for the entire variation. Indeed, at 5 cmH_2_O it is more difficult, even for trained personnel, to discriminate between parenchyma and pleural effusion, a process that is easier at 45 cmH_2_O. Therefore, manual segmentation is intrinsically associated with some degree of inaccuracy. Interestingly, as shown in Table 3, AI had the same degree of inaccuracy and closely mimicked the manual segmentation. Moreover, the more severe the ARDS, i.e., the extent of the densities and pleural effusions that are is present in approximately 80% of ARDS patients (Gattinoni et al., 1986), the greater the probability of inaccuracy. As opposed to the CT images of the training set, the lungs of the patients in the test CT were more poorly segmented, which may indicate a slight overfitting of the CNNs.

The most relevant quantitative CT variables that may have an impact on clinical management are the recruitability and the volume of the lung open to the gas. The latter is frequently referred to as “baby lung,” since in ARDS its size may resemble that of a 3-year-old child. The baby lung is represented by the amount of normally aerated tissue, which conventionally includes the voxels between −500 and −900 HUs (Gattinoni et al., 1986). AI-segmentation performed remarkably well under this definition, with an overall agreement within few grams. Knowledge of the baby lung and its associated gas volume will allow a straightforward measurement of the strain occurring during mechanical ventilation. Determining the strain, i.e., the ratio of tidal volume plus PEEP volume to the FRC, is a fundamental information when setting the ventilator, since excessive strain is a primary cause of ventilation-induced lung injury (Chiumello et al., 2008).

The recruitability can be estimated either by assessing the amount of lung tissue which regains aeration, or by measuring the increase in the size of the baby lung when the airway pressure is increased. This allows the normal aeration of pulmonary units, which were previously collapsed or simply poorly inflated. Measuring recruitment as a non-aerated tissue fraction difference had a bias of +0.3% (CI: +6.2/−5.5%) on the test set. We believe, from a clinical standpoint, that these numbers are more than adequate to define the recruitability, which is usually roughly defined as a binary variable, i.e., the patient is either a “recruiter” or a “non-recruiter.” A more precise definition of recruitability, which may range from 0% to more than 50% of the total lung mass, that would be easily clinically available with AI-segmentation, may represent an important step ahead when tailoring mechanical ventilation or setting PEEP. When we defined recruitment as changes in the baby lung dimensions, AI performed extremely well compared with manual segmentation.

Most of the advances on our pathophysiological understanding of ARDS derive from the quantitative CT scan analysis. An easy availability of the CT-qa may play an important role in setting a proper ventilation and, maybe more importantly, to avoid harmful approaches.

## Limitations

In the original U-Net, the original input image is first convoluted with 64 filters. Due to hardware limitations, this was not possible with our U-Net, so we started with 32 convolution filters. We have found that in the edge areas of the lungs, especially with very badly damaged lung tissue, segmentation is much worse. In addition, the lung CTs unknown to the model are segmented more poorly than the trained lung CTs, which indicates on the one hand that the Network is slightly overfitted, or on the other hand, that training was carried out with too few lung CT variations. Our ANN was developed with NI-LabVIEW, NI-Vision and the Deep Learning Toolkit from Ngene (DeepLTK). These are all commercial, license-protected software products. This means that an application cannot be used freely. The DeepLTK still has a number of limitations: IOU and Dice coefficient are the only metrics so far. More will be added in the next releases. Shape quality performance metrics like ASSD or BF-Score are not yet supported. The only optimization algorithm is Stochastic gradient descent. Further algorithms such as Adam, Adagrad, AdaDelta, RMSProp, and Nesterov are being developed for the next releases. So far, Mean Squared Error is the only loss type for 3D data. Cross Entropy loss is currently only available for 1D data. But it will be available for 3D data in the next update. It is a specific of DeepLTK toolkit (at the moment) that the complete dataset is preloaded on the CPU RAM (as a 4D single precision floating point array) to speed up miniBatch fetching and feeding to the Network for the training process. As during the training process, the whole dataset will be utilized for several (hundreds of) epochs, it is reasonable to preload decoded dataset and store it on the RAM to speed up the training process. Loading a miniBatch of data from disk is also reasonable, in case of large datasets, and at the cost of speed, but currently it is not implemented in the toolkit. A 3D semantic segmentation architecture is still not possible with the DeepLTK.

## Conclusion

The trained model based on the U-Net can automatically segment the lungs in the CT with the limitations mentioned. The automatic segmentation of a full lung CT scan with approximately 100 sections with a slice thickness of 5.0 mm took approximately 5 s, compared to manual segmentation which can take up to an hour. Due to the still poor performance compared to Python-based CNNs, we plan to further improve the U-Net developed with LabVIEW and optimize it for the detection of differently damaged lung areas. We are convinced that the widest possible variety of different lung pathologies and CT reconstruction parameters can significantly improve a suitable segmentation CNN. Therefore, we will increase the amount of input data through augmentation by varying the brightness, contrast, gamma and grain of the CT images and applying them to an increasing number and variety of lung pathologies. We plan to further modify the Network architecture through tests, while changing the miniBatch size, varying the probability of dropout layers, and varying the training parameters (i.e., optimizer, loss type, momentum, weight decay, and training type) are interventions for future research and further improvement. This should widen the field of potential applications and increase the already convincing validity of image data processing. In order to play with all these possibilities, one will require greatly advanced hardware with much better performance compared with the hardware used for this study.

The development of a reliable clinical diagnostic system, able to perform the automatic detection and consecutively the quantitative analysis of lung tissues immediately after performance of a lung CT scan seems conceivable and also practicable. Such a tool would have significant impact on diagnosing and selecting the appropriate therapeutic interventions for each individual patient suffering from severe lung injury.

## Data Availability Statement

The raw data supporting the conclusions of this article will be made available by the authors, without undue reservation.

## Author Contributions

PH: study design, programming of the ANN, data collection, data analysis, figure design, and writing. MB: data analysis, statistical interpretation, figure design, and writing. MC: data collection, data interpretation, and image processing. LS, OM, KM, MQ, and LG: study design, data analysis, data interpretation, and writing. JL: data analysis, data interpretation, and revision of the manuscript. All authors critically revised and accepted the manuscript in its current form.

## Conflict of Interest

The authors declare that the research was conducted in the absence of any commercial or financial relationships that could be construed as a potential conflict of interest.

## Publisher’s Note

All claims expressed in this article are solely those of the authors and do not necessarily represent those of their affiliated organizations, or those of the publisher, the editors and the reviewers. Any product that may be evaluated in this article, or claim that may be made by its manufacturer, is not guaranteed or endorsed by the publisher.
