# Supplementary material for: Using Artificial Intelligence for Automatic Segmentation of CT Lung Images in Acute Respiratory Distress Syndrome
Source: Front Physiol. 2021 Sep 14;12:676118. doi: 10.3389/fphys.2021.676118 (PMC8476971; doi:10.3389/fphys.2021.676118)
Supplement: Supplementary file 1 [file Data_Sheet_1.docx]

**Using Artificial Intelligence for Automatic Segmentation of CT lung Images in Acute Respiratory Distress Syndrome**

**Peter Herrmann^1^, MSc, Mattia Busana^1^, M. D, Massimo Cressoni^4^, M. D, Joachim Lotz^2^, Onnen Moerer^1^, Leif Saager^1^, M. D, Konrad Meissner^1^, M. D, Michael Quintel^1,3^, M. D, Luciano Gattinoni^1^, M. D. FRCP**

^1^Department of Anesthesiology, University Medical Center Göttingen, Göttingen, Germany ^2^Institute for Diagnostic and Interventional Radiology, University Medical Center Göttingen, Göttingen, Germany ^3^Department of Anesthesiology, Donau-Isar-Klinikum Deggendorf, Deggendorf, Germany ^4^Unit of Radiology, IRCCS Policlinico San Donato. San Donato, Milan, Italy

## **Online Supplement**

In the online supplement we provide additional selected images for the automatic segmentation of test and training CTs. The figures E1 and E2 show examples of each series of trained and tested CT images of selected patients. The intersection over union value in the trained CT images is worse, the more damaged the lung is and the nearer the edge the lung layer is (IoU ~ 70%). In the trained CT images even in the most peripheral areas of extremely damaged lungs, the calculated IoU values were > 95%.


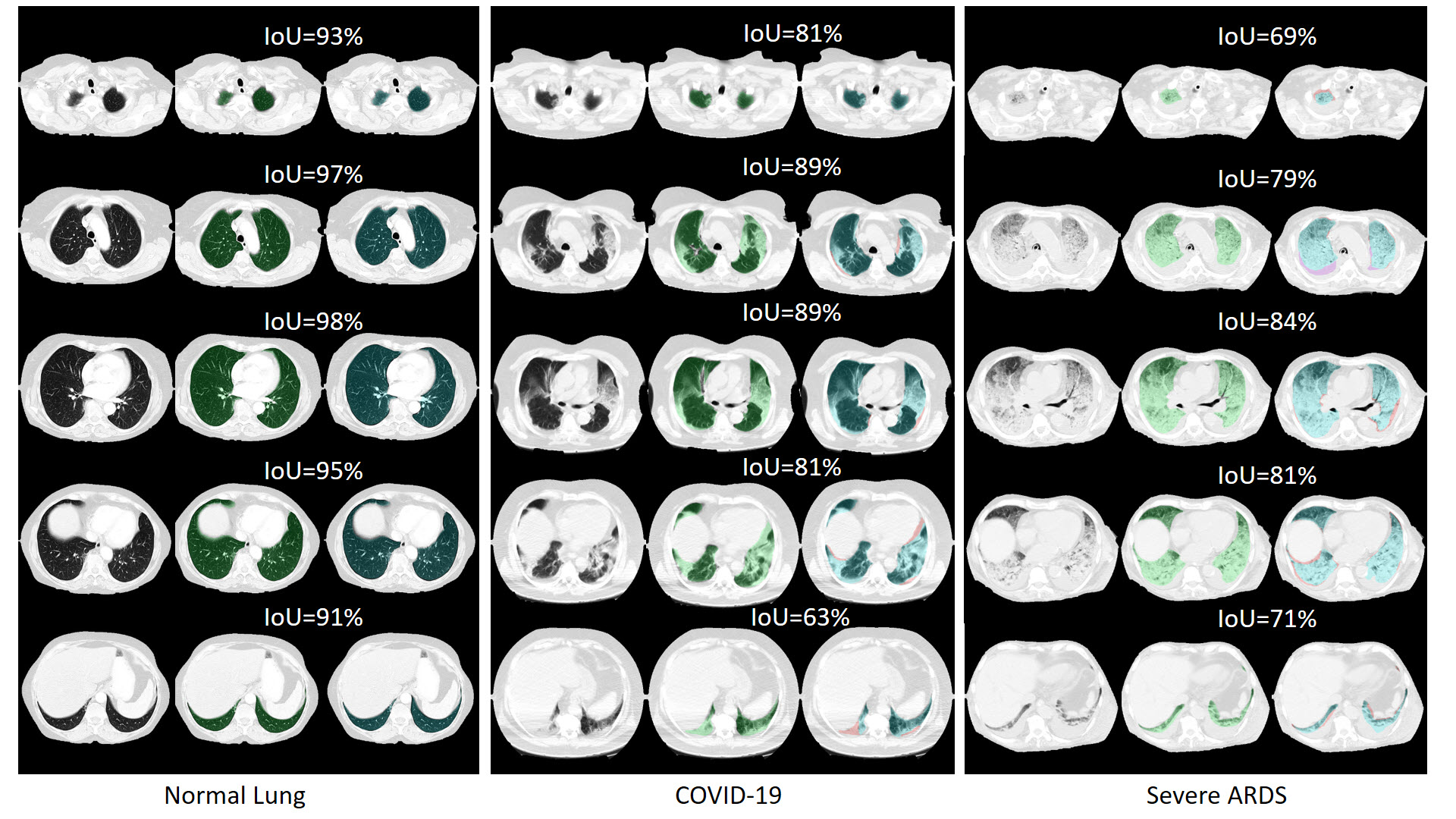


**Figure E1**: Series of tested lung CT images from selected patients with healthy lungs, COVID-19, and severe ARDS. From top to bottom, slices from apex to base, from left to right original CT, green overlay = manual segmentation (ground truth), blue overlay = automatic segmentation (prediction). Red areas are unrecognized areas; purple areas are incorrectly recognized areas.


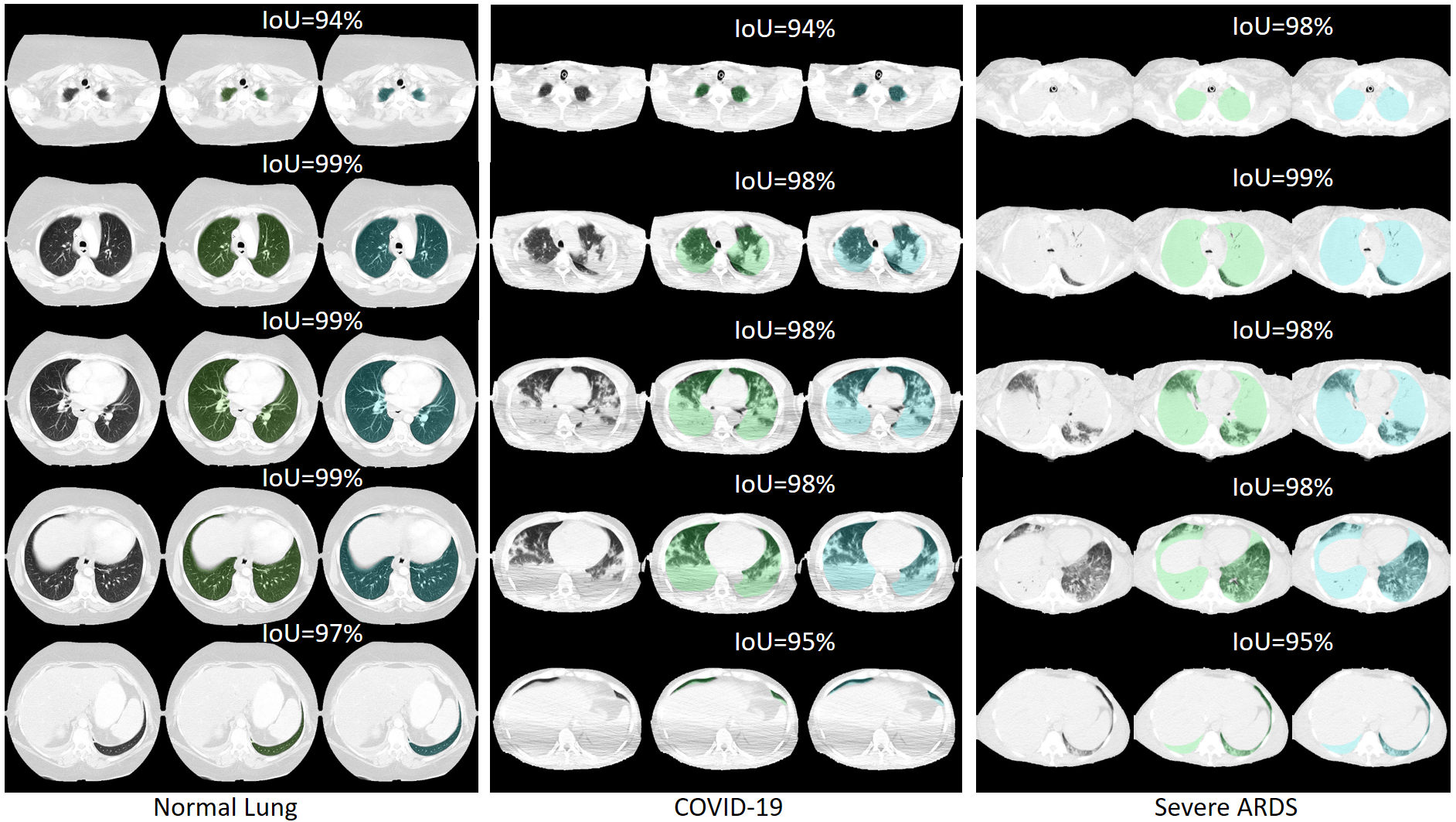


**Figure E2:** Series of trained lung CT images from selected patients with healthy lungs, COVID-19, and severe ARDS. From top to bottom, slices from apex to base, from left to right original CT, green overlay = manual segmentation (ground truth), blue overlay = automatic segmentation (prediction). We selected three patients with impressively pathological lung changes. A patient with undamaged lungs, a patient with pronounced COVID-19 disease and a patient with severely injured ARDS lungs.

In the coronal sectional image (figure E3), the peripheral regions in which lung segmentation is worse can be seen better.


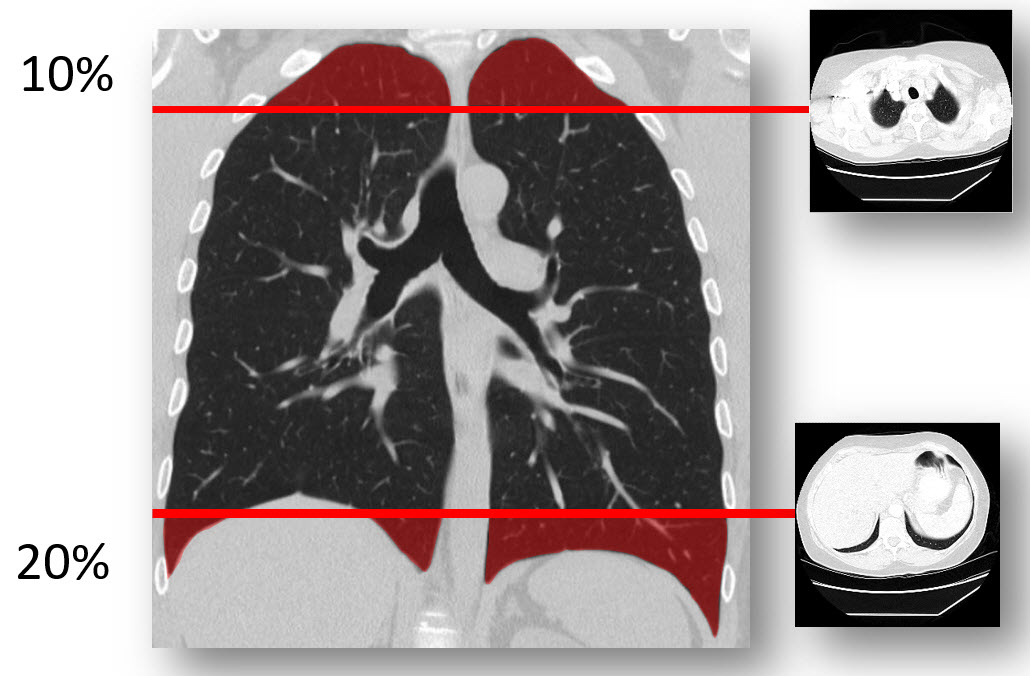


**Figure E3**: Edge regions of the lungs in which the segmentation functions worse in the test images and in severely damaged lung tissue.

Figure E4 and E5 show tested lung CTs of two sample patients with ARDS at Peep 5. Overdistended, normally ventilated, poorly ventilated and non-ventilated areas are color-coded. (Overdistended = blue, normally ventilated = green, poorly ventilated = orange and not ventilated = red).


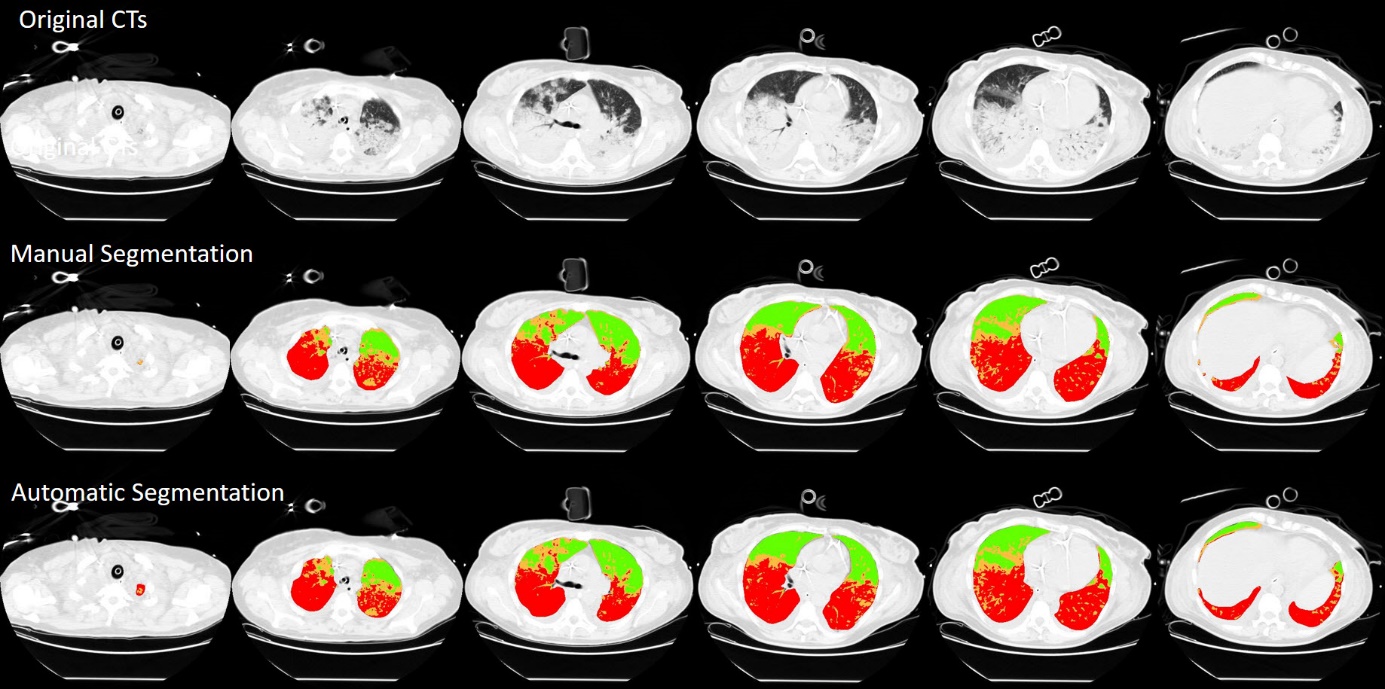


**Figure E4**: tested lung CTs of sample patient 1.

Segmentation in sample patient 1 worked very well, and shows a difference in the tissue mass of only 1.8%. In contrast, too much damaged tissue is detected in the CTs of sample patient 2. Accordingly, in this case the proportion of tissue mass is 6.1% higher with automatic segmentation than with manual segmentation.


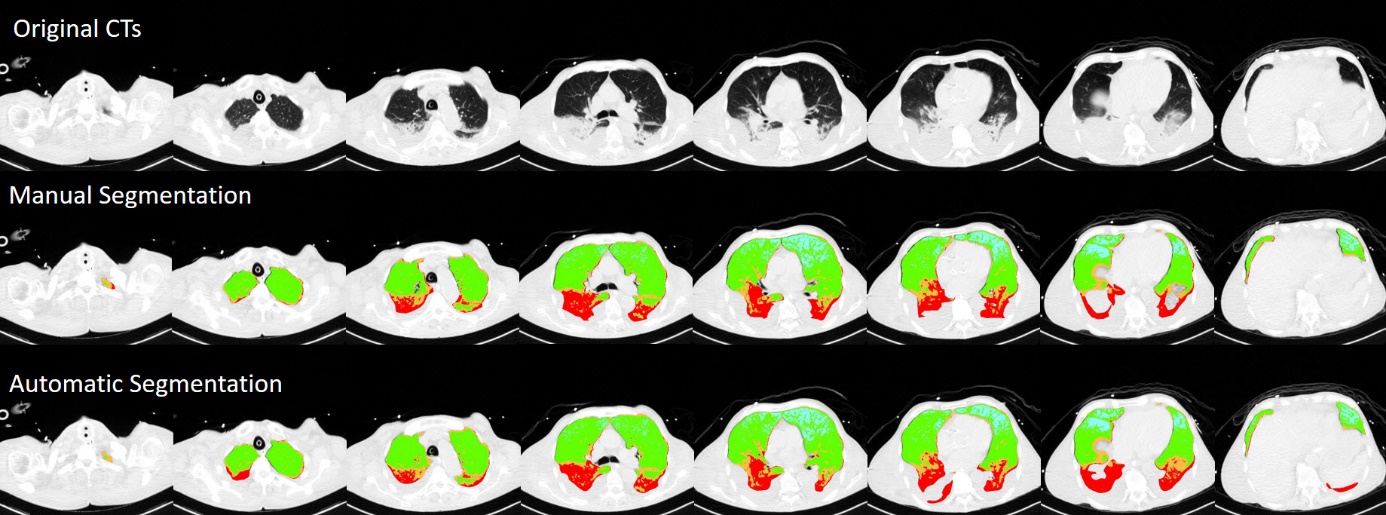


**Figure E5**: tested lung CTs of sample patient 2.

**
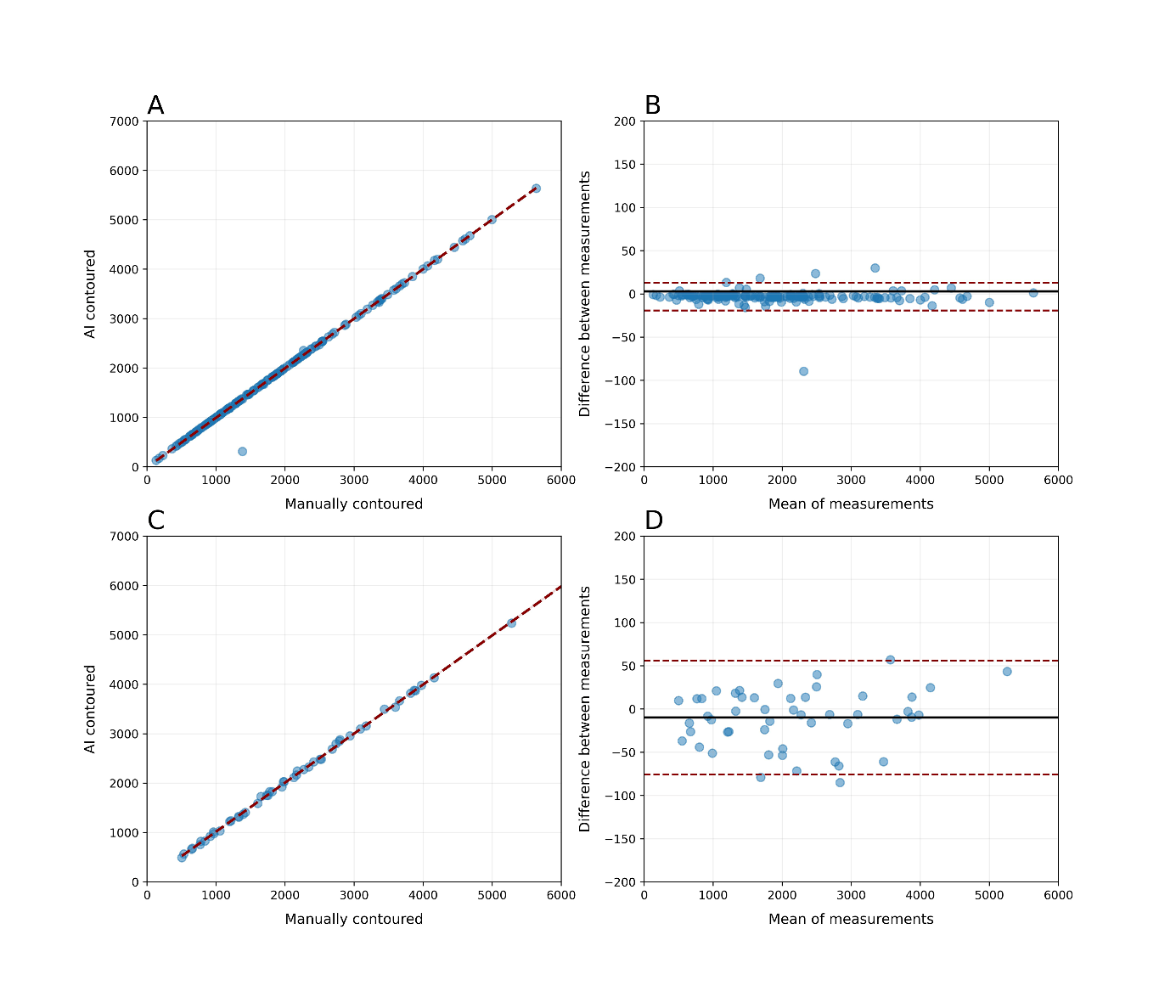
**

**Figure E6:** Linear regression (panel A) and Bland Altman analysis (panel B) of the agreement between manual and AI-segmentation on training set when CT-qa was used to measure the total lung volume. The R2 of the regression (x axis = manual segmentation, y axis = AI-segmentation) was 0.99. The bias between the two measurements was -3.1 ml [CI +13.0 /-19.1]. The same analysis (panel C and D) is repeated on the test set. The R2 of the regression was 0.99 and the bias -9.8 ml [CI: +56.0 / -75.7 ml].


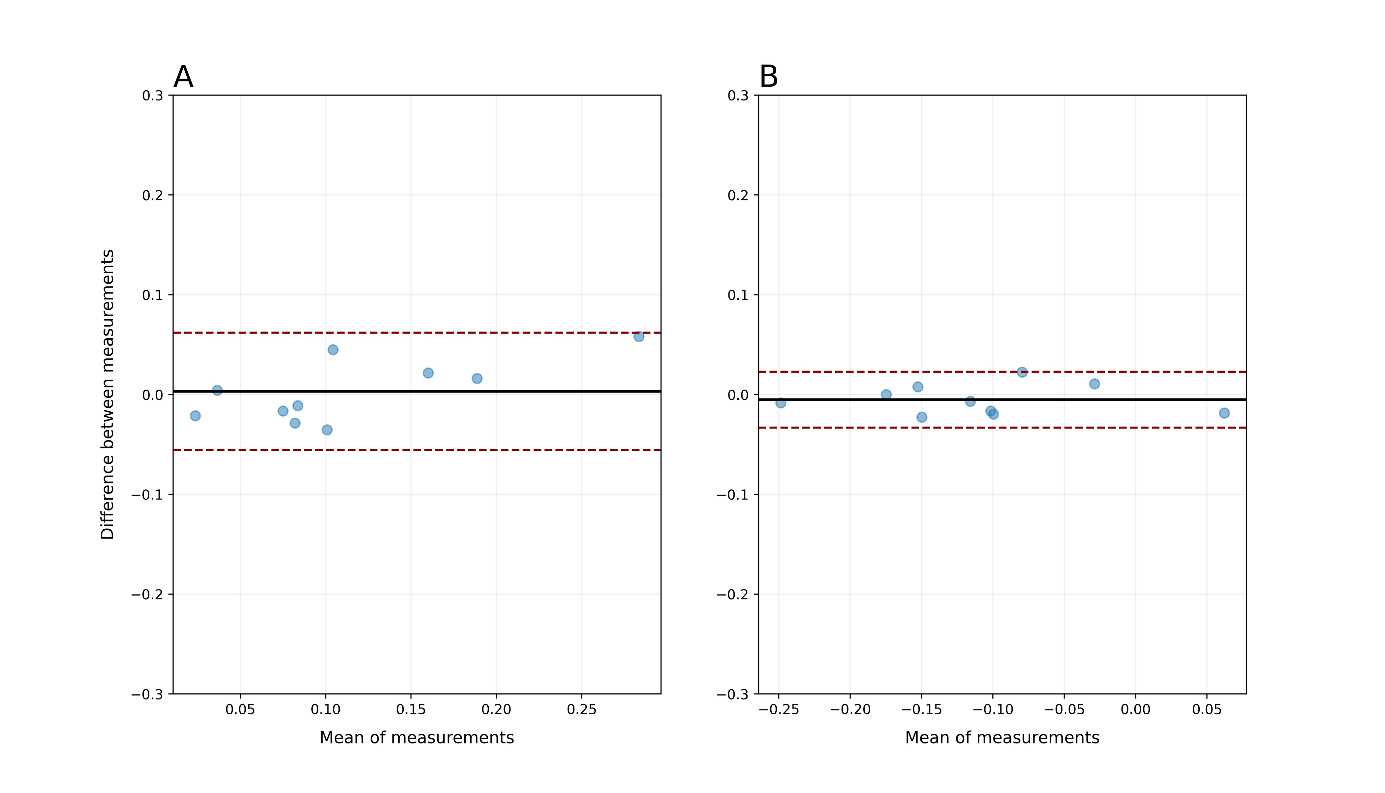


**Figure E7:** Panel A: Bland Altman analysis of the recruitment fraction (expressed as the variation of non-aerated tissue fraction between 5 and 45 cmH2O) on the test set. The bias between the two measurements was +0.3% [CI: +6.2/-5.5%]. -0.5% [CI: +2.3/-3.3%]. Panel B: Bland Altman analysis of the recruitment fraction (expressed as the variation of the well-aerated tissue fraction between 5 and 45 cmH2O) on the test set. The bias between the two measurements was -0.5% [CI: +2.3/-3.3%].
